# Efficiency, error and yield in light-directed maskless synthesis of DNA microarrays

**DOI:** 10.1186/1477-3155-9-57

**Published:** 2011-12-08

**Authors:** Christy Agbavwe, Changhan Kim, DongGee Hong, Kurt Heinrich, Tao Wang, Mark M Somoza

**Affiliations:** 1 Institute of Inorganic Chemistry, University of Vienna, Währinger Strasse 42, A-1090 Vienna, Austria; 2 Center for Nanotechnology, Department of Electrical and Computer Engineering, University of Wisconsin, Madison WI 53706, USA; 3 Center for Nano Science and Technology, Department of Electrical Engineering, University of Notre Dame, Notre Dame, IN 46556, USA; 4 Current Address: Intel Corporation, 8000 S. Federal Way, Boise ID 83716, USA

**Keywords:** Microarray, phosphoramidite chemistry, NPPOC, gene synthesis

## Abstract

**Background:**

Light-directed *in situ *synthesis of DNA microarrays using computer-controlled projection from a digital micromirror device--maskless array synthesis (MAS)--has proved to be successful at both commercial and laboratory scales. The chemical synthetic cycle in MAS is quite similar to that of conventional solid-phase synthesis of oligonucleotides, but the complexity of microarrays and unique synthesis kinetics on the glass substrate require a careful tuning of parameters and unique modifications to the synthesis cycle to obtain optimal deprotection and phosphoramidite coupling. In addition, unintended deprotection due to scattering and diffraction introduce insertion errors that contribute significantly to the overall error rate.

**Results:**

Stepwise phosphoramidite coupling yields have been greatly improved and are now comparable to those obtained in solid phase synthesis of oligonucleotides. Extended chemical exposure in the synthesis of complex, long oligonucleotide arrays result in lower--but still high--final average yields which approach 99%. The new synthesis chemistry includes elimination of the standard oxidation until the final step, and improved coupling and light deprotection. Coupling Insertions due to stray light are the limiting factor in sequence quality for oligonucleotide synthesis for gene assembly. Diffraction and local flare are by far the largest contributors to loss of optical contrast.

**Conclusions:**

Maskless array synthesis is an efficient and versatile method for synthesizing high density arrays of long oligonucleotides for hybridization- and other molecular binding-based experiments. For applications requiring high sequence purity, such as gene assembly, diffraction and flare remain significant obstacles, but can be significantly reduced with straightforward experimental strategies.

## Background

**Figure 1:**
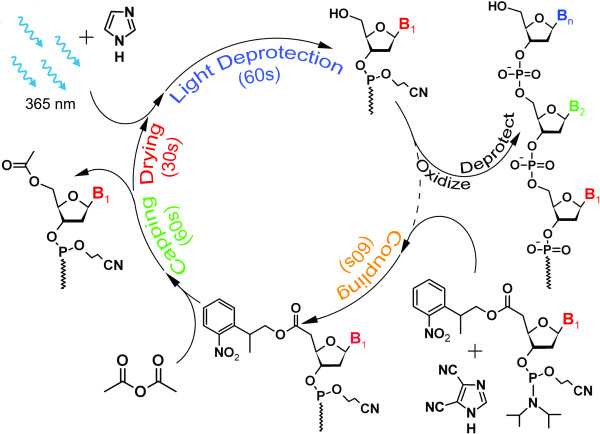
**Phosphoramidite synthesis cycle in maskless, light-directed synthesis of microarrays**. The cycle is similar to that used in solid-phase synthesis of nucleic acids with some key differences: UV light from the I-line of mercury, in the presence of an organic base, is used to deprotect the 5'-OH; the array surface is dried with helium before photodeprotection; oxidation of the phosphites is not required in the cycle because they are not exposed to acid; the final chemical deprotection must not cleave the nucleic acids from the surface. The duration of each step in the synthesis cycle depends on experimental conditions and objectives, but typical values are given.

*In situ*, light-directed synthesis of microarrays is an extension of photolithographic technology from the semiconductor industry combined with combinatorial chemistry of phosphoramidites with a photolabile 5'-hydroxyl protecting group [1]. The original application of this technology lead to the foundation of Affymetrix in 1991. Affymetrix microarrays are manufactured using light exposure patterned by physical masks placed over the synthesis surface. A similar process, but one that avoids the need for the expensive and time-consuming synthesis of the large numbers of the photolithographic masks required for each microarray design [2] lead to the foundation of NimbleGen in 1999. Maskless array synthesis (MAS) uses a digital micromirror device (DMD) in place of photomasks to deliver patterned ultraviolet light. The pattern displayed on the micromirror device is transferred to the synthesis surface, where the array layout and oligonucleotide sequences are determined by selective removal of the photocleavable protecting groups on the 5'-end of the terminal phosphoramidites on the microarray. The phosphoramidite synthesis cycle in MAS is illustrated in Figure 1. Reagent delivery to the array surface is accomplished with a slightly modified oligonucleotide synthesizer, i.e., an Expedite 8909 or equivalent system. The display of virtual masks on the DMD is coordinated with the chemical delivery by an external computer.

From an experimental perspective, the MAS tool is a convenient platform for developing and synthesizing new microarrays. It is a highly robust desktop system with few moving parts and very modest maintenance requirements. The only required external inputs are bitmap files representing the virtual masks as well as other files for guiding synthesis, readily available chemical consumables, and the substrate. Maskless microarray synthesis is also very flexible, can be optimized for a variety of applications, and has a fast turn-around time. Within the XGA (eXtended Graphics Array) pixel limitations of the DMD--1024 × 768 micromirrors (or more recently, standard high-definition, 1920 × 1080)--between 1 and 786 432 sequences can be simultaneously synthesized with flexible layout. The chemistry can also be modified to suit the experimental design. The most common use of MAS is for DNA oligonucleotide microarray synthesis, but has been extended to RNA [3,4] and to peptide microarray synthesis [5,6]. The microarrays are typically synthesized on standard-format glass microscope slides, but other chemically-compatible substrates with native surface hydroxyl groups, or surfaces which can be functionalized to add hydroxyl groups, can be used. Glassy carbon and nanocrystalline diamond films [7], carbon-on-metal films [8], and electron beam patterned hydrogen silsesquioxane [9] have been successfully used as substrates in MAS.

Compared to conventional solid-phase synthesis of oligonucleotides, which has reached stepwise coupling efficiencies of ~99% over several decades of optimization [10,11], lower yields in MAS are a significant obstacle to long or high-fidelity oligonucleotide synthesis. The optimum oligonucleotide length for microarrays depends strongly on the application. For typical hybridization-based experiments such as gene expression analysis, longer probes result in higher sensitivity but lower specificity. The optimum length depends on multiple design parameters, including the number of probes per gene, but longer probe lengths are favored in the literature [12-14]. For commercially synthesized microarrays, probe length varies from 25 to 70 bases, with 60 bases as the most common length [15]. Even though microarray manufacturers can compensate for lower coupling efficiencies by shortening probe length and increasing the number of probes per gene, better coupling lowers costs and allows increased genome coverage. Microarrays used for profiling DNA-binding molecules contain self-complementary sequences with a total length of ~40 bases [16-18]. A significantly more demanding application is *de novo *gene synthesis from microarray-derived oligonucleotides. Microarrays are a very cost-effective means to generate the necessary pool of hundreds to thousands of oligonucleotides required for gene assembly [19], but the low yield of correct sequences requires significant error correction methods [20-22].

In conventional solid-phase synthesis, the principle sources of sequence error are failed couplings due to coupling efficiencies less than 100%, and depurination due to cumulative exposure of purines to the acidic detritylation conditions [23]. Errors arising from the former type of failure can be mitigated by acetylating (capping) the residual 5'-hydroxyl groups during synthesis, which prevents further chain growth and facilitates purification on the basis of chain length. Abasic sites from depurination events lead to strand cleavage during the nucleobase deprotection step, which also enables length-based purification. A coupling failure on the last cycle, a coupling failure followed by a capping failure, or a 5'-deprotection failure, leads to a single-base deletion, which is the dominant error in artificial gene constructs [21]. The sources of sequence error in MAS are more complex. Deletion errors are somewhat more common due to slightly lower coupling efficiency, but in addition, insertion errors contribute significantly to the overall error rate. Insertion error occurs when unavoidable imperfections in the optical imaging system results in unintended exposure on the microarray. Image drift, scattering, diffraction and flare all contribute to insertion error. The effect of stray light on the fidelity of photo-lithographically synthesized microarrays was first discussed by Garland *et al *in the context of arrays produced using photo-generated acids [24]. Here we report on recent experimental results and simulations of synthesis error in MAS, as well as methods to optimize sequence quality for microarrays intended for both hybridization-based experiments and oligonucleotide synthesis for gene assembly.

## Results

### Coupling efficiency

In MAS, there are two potential factors that contribute to deletion errors, low stepwise coupling efficiency of the phosphoramidites and incomplete NPPOC removal. NPPOC removal will be considered separately, in the section on exposure. There are two methods for investigating coupling efficiency in light-directed synthesis, terminal-labeling studies and sequencing. We use terminal labeling as the primary tool for measuring and optimizing coupling efficiency because the results are obtained immediately following synthesis and are simple to interpret, but sequencing data provides an independent confirmation of the results.

**Figure 2:**
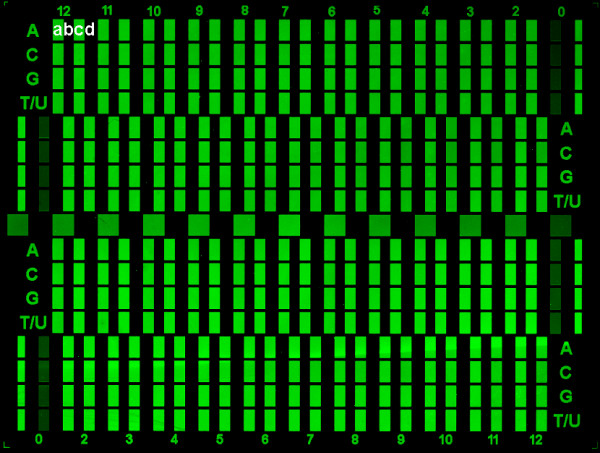
**Scan image of 4-plex microarray used for coupling studies**. Coupling yield based on up to four sets of coupling parameters (or in a separate experiment, different phosphoramidites) were determined on single microarrays. The labels A, C, G, and T/U can refer to the four amidites, but in this scan only NPPOC-dT was used with four different sets of coupling parameters. The numbers at the top and bottom label the length of the experimental oligonucleotide, which is synthesized after a thymidine 15mer linker. Each numerical label is associated with four features, labeled in white in the top left corner: Cy3-labeled *n*-mer (a), unlabeled *n*-mer (b), Cy3-labeled 1-mer (c), and unlabeled 1-mer (d). The normalized intensity I of each *n*-mer is calculated as [I(a)-I(b)]/[I(c)-I(d)]. The data from this array is given in Table 2, Array 4. The 0-mer features are the result of coupling from a phosphoramidite port on the synthesizer containing pure acetonitrile and are used to calibrate the capping efficiency. Spot intensities are uniformly high due to high coupling efficiency.

Sequences of the form (substrate)- 3'-T_15_-X_*n*_-T_2_-(Cy3/no_Cy3)-5', X = A, C, G, or T and *n *= 0 to 12 were synthesized onto the arrays using capping after each coupling reaction to terminate failed sequence additions. Half of the feature corresponding to each coupling number was terminally labeled with Cy3 and the other half was not labeled and used to determine the background fluorescence due to non-specific binding of the dye to the oligonucleotide (cf. ref. [25]). An example of a microarray used to measure coupling efficiency is shown in Figure 2. The 4-plex design allows the simultaneous measurement of the coupling efficiency of four phosphoramidites, or in a separate experiment, of four coupling protocols for a single phosphoramidite. Each numbered rectangular feature X_*n *_has four sub-features, labeled in the white letters (a, b, c, d) in Figure 2, that are used to calculate the coupling efficiency: (a) X_*n*_+Cy3, (b) X_1_+Cy3, (c) X_*n*_, (d) X_1_. The coupling efficiency is determined by measuring the fluorescence intensity (I) of each and fitting the twelve points f(*n*) = [I(X_*n*_+Cy3)-I(X_*n*_)]/[I(X_1_+Cy3)-I(X_1_)] with a two parameter single exponential decay, *f*(*s*) = *A*e^-*bs*^, where *b *is the fluorescence decay constant and the average percent yield for all the couplings is 100% (1-*b*). The labeled features in Figure 2 appear uniformly bright due to the high coupling efficiency in this experiment (> 99%). The unlabeled controls also appear uniformly dark in this Figure, but the numerical data shows that the non-specific binding of the Cy3 phosphoramidite increases with increasing oligonucleotide length. Fluorescent intensity is measured as the average pixel intensity of the corresponding feature in the scanned image of the microarray.

After optimizing synthesis conditions (see below) using NPPOC-dT, the coupling efficiency of all four NPPOC phosphoramidites was tested simultaneously on single microarrays. The synthesis parameters were: a single final oxidation step, 30 s helium drying after capping, 60 s coupling using the pulse sequence in Table 1, 0.025 M amidite in 0.25 M DCI activator, exposure dose of 12 J/cm^2^, and 120 s acetic anhydride capping. For this experiment, the size of each feature on the microarray was made smaller (25 in 36 mirror layout) and the position of each of the hundreds of replicates of each feature was randomized across the array surface. In addition, a short segment with sequence GAAAA was synthesized between the terminus of each experimental *n*-mer sequence and the terminal Cy3 to reduce the sequence-dependence of the fluorescence intensity of Cy3 [26]. The measured coupling efficiencies were: 99.8% (dA), 98.0% (dC), 98.6% (dG), 99.4% (dT), and 99.0% (global fit of all four base data).

**Table 1:** Synthesis protocols

**Cycle T**					**Cycle M (Cy3)**				
	
**Function**	**Mode**	**pulse**	**sec**	**Description**	**Function**	**Mode**	**pulses**	**sec**	**Description**
	
**$Coupling**					**$Coupling**				
1/*Wsh	*/PULSE	20	0	"Flush system with Wsh"	1/*Wsh	*/PULSE	20	0	"Flush system with Wsh"
2/*Act	*/PULSE	6	0	"Act"	2/*Act	*/PULSE	6	0	"Act"
21/*T + Act	*/PULSE	5	0	"T + Act"	25/*8 + Act	*/PULSE	5	0	"8 + Act"
2/*Act	*/PULSE	8	0	"Chase with Act"	2/*Act	*/PULSE	8	0	"Chase with Act"
1/*Wsh	*/PULSE	3	60	"Couple monomer"	1/*Wsh	*/PULSE	3	300	"Couple monomer"
1/*Wsh	*/PULSE	10	0	"Flush system with Wsh"	1/*Wsh	*/PULSE	20	0	"Flush system with Wsh"
**$Capping**					1/*Wsh	*/PULSE	10	0	"Flush system with Wsh"
13/*Caps	*/PULSE	10	0	"Caps"	2/*Act	*/PULSE	6	0	"Act"
13/*Caps	*/PULSE	10	120	"Caps"	25/*8 + Act	*/PULSE	5	0	"8 + Act"
12/*Wsh A	*/PULSE	25	60	"Wsh A"	2/*Act	*/PULSE	8	0	"Chase with Act"
12/*Wsh A	*/PULSE	30	0	"Wsh A"	1/*Wsh	*/PULSE	3	300	"Couple monomer"
40/*Gas A	*/PULSE	1	30	"Dry column"	1/*Wsh	*/PULSE	10	0	"Flush system with Wsh"
**$Oxidizing**					**$Oxidizing**				
15/*Ox	*/PULSE	30	0	"Oxidize"	15/*Ox	*/PULSE	30	0	"Oxidize"
12/*Wsh A	*/PULSE	45	0	"Flush system with Wsh A"	12/*Wsh A	*/PULSE	300	0	"Wsh A"
17/*Aux	*/PULSE	45	0	"Aux"	12/*Wsh A	*/PULSE	300	300	"Wsh A"
130/*Event 2 Out	*/NA	4	3	"Event 2 Out"					
17/*Aux	*/PULSE	16	60	"Aux"					
12/*Wsh A	*/PULSE	25	0	"Flush system with Wsh A"					

Generic chemical synthesis protocol (Expedite 8909) for NPPOC-dT coupling (representative of all four bases) and for the terminal label coupling. The 5'-NPPOC light-deprotection step takes place during the "Event Out". During the light exposure, NPPOC removal is aided by washing with exposure solvent from the "Auxiliary" port. If no capping is used, only the Gas A step is retained under the $Capping heading. The oxidation step (15/*Ox) is often omitted for DNA phosphoramidite couplings.

Sequencing of microarray eluted oligonucleotides was also used as an independent measurement of coupling efficiency. With a mixed-base 40-mer synthesized on a microarray using a 1 base/4 cycle protocol, an average stepwise coupling efficiency of 98.5% was calculated from the sequence data. The 1 base/4 cycle protocol, in which the elongation of all microarray oligonucleotide sequence by 1 base, requires 4 cycles (light exposure, coupling, capping)--one for each of the 4 DNA bases--is the normal synthesis cycle in MAS. The 1 base/4 cycle protocol leads to the maximum exposure of microarray oligonucleotides to reagents and stray light.

### Coupling times

**Figure 3:**
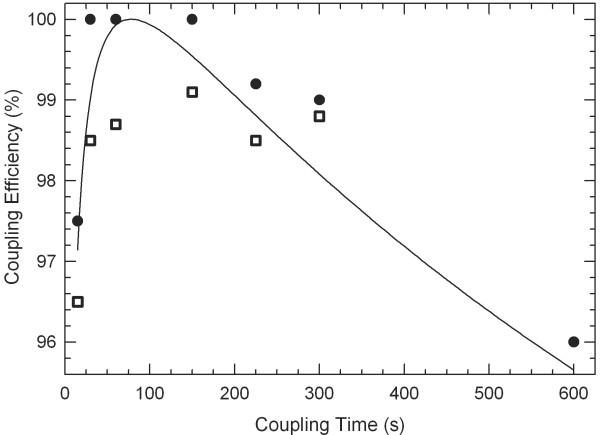
**Coupling efficiency vs. time of NPPOC-dT phosphoramidite**. Coupling efficiency starts at ~95% at the lowest possible transit time, increases to a maximum and then decreases slowly for long coupling times. Circles indicate a phosphoramidite concentration of 0.03 M. At this concentration and between 30 s and 150 s coupling times, no yield differences can be observed between 1-mers and 12-mers. Squares show coupling efficiency data for the phosphoramidite diluted to 75% of the original concentration. The curve serves as a guide to the eye (lognormal; μ = 52; σ = 6.9; scaled to 100% at the maximum at 80 sec.).

Figure 3 shows the effect of coupling time on coupling efficiency using NPPOC-dT as a representative phosphoramidite. Using the standard concentration of 0.03 M dT in 0.25 M DCI activator, the yield of 12mers cannot be distinguished from that of 1mers for coupling times between 30 and 150 seconds. At shorter times, the yield drops quickly to a minimum (not shown) of ~95% for a coupling time of ~1 second, the approximate minimum possible amidite residence time in the reaction cell. At longer coupling times, the yield drops slowly, suggesting that prolonged exposure to activator cleaves DNA strands in a length-dependent manner or the amidite itself is degraded by the activator. These results are based on simple one-chip one-experiment microarrays. Repeating the experiments using the 4-plex-type coupling microarrays shown in Figure 2, and using a mix of short and long coupling times for different length series on the same array, results in similar but slightly better yields for the long coupling times (300 s) versus short coupling times (50 s). This result favors the hypothesis that long coupling times increase stepwise coupling yields but result in lower overall yield due to cumulative chemical damage to the microarray. The primary consequence of this result is that the optimal coupling time is dependent on the complexity of the microarray, with long oligonucleotide microarrays favoring shorter coupling times. Similarly, the overall yield will be lower for long oligonucleotide arrays. The effective stepwise synthesis yield in microarrays based on amidites with lower coupling efficiency, such as RNA NPPOC amidites [3,4], may be limited to < ~97% because the longer coupling times needed for such amidites is limited by considerations of overall array degradation.

### Oxidation

**Figure 4:**
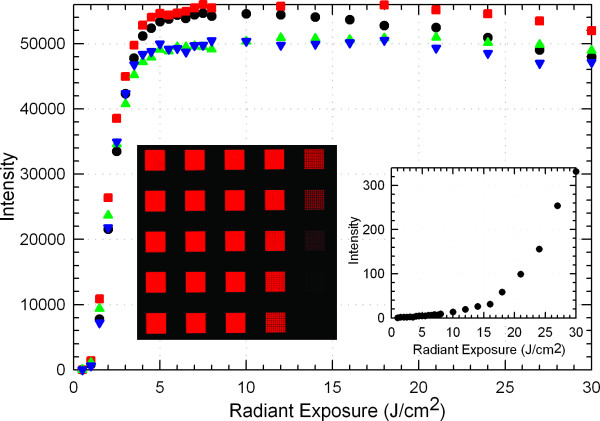
**Single sequence exposure gradients with different oxidation protocols**. Signal intensity is from a hybridized, Cy3-labeled complementary sequence. Black circles: single oxidation after last coupling. Red squares: an oxidation at the middle and one at the end. Green triangles: oxidation after every fourth coupling and after the final coupling. Blue downwards triangles: Oxidation after every base. Insert graph: no oxidation steps. Inset images: one of four replicate gradients on the array with a middle and terminal oxidation; exposure increases bottom to top and right to left.

An oxidation step is required after every coupling in solid-phase synthesis of oligonucleotides because the acid used for 5'-deprotection (detritylation) would otherwise cleave the DNA backbone at the phosphite. Unnecessary oxidation may reduce yield by introducing water into the system, which reacts with the activated amidites. In addition, the iodine may promote guanine modifications prone to depurination [27]. We conducted two types of experiment to determine how much oxidation is optimal in MAS. First we synthesized simple microarrays with a single 25mer sequence (5'-GTC ATC ATC ATG AAC CAC CCT GGT C-3') on an light exposure gradient--individual features on the array receive from 0 to 30 J/cm^2^--and each separate microarray subject to a different oxidation protocol. After synthesis and deprotection, the microarrays were deprotected and hybridized with the 5'-Cy3-labelled complementary sequence and scanned. The results are shown in Figure 4. The two microarrays subjected to the least amount of oxidation, a single oxidation after the last coupling and a double oxidation (one in the middle and once after the last coupling) have a significantly higher hybridization signal than the two microarrays with the most oxidizer exposure, i.e. oxidation after every coupling and oxidation after every fourth coupling plus a final oxidation. Microarrays synthesized without any oxidation are mostly destroyed during deprotection as shown in the Figure 4 inset. The shape of the curves and the optimal exposure will be discussed in the Exposure section. We also used terminally-labeled microarrays, like the 4-plex array in Figure 2, to measure the stepwise coupling efficiency with and without oxidation. Using an otherwise identical protocol and an exposure of 12 J/cm^2 ^array features subject to oxidation after every coupling had a 0.2% lower average stepwise yield than features with only a single final oxidation. This value is consistent with the hybridization based values since it corresponds to a final yield difference of correct sequence 25mers of about 5%.

### Helium drying

The MAS protocol includes a step between coupling and light deprotection in which helium is used to blow out the acetonitrile wash and dry the substrate surface--"helium blow". There is no obvious physical or chemical relevance to this step, but it significantly increases the stepwise coupling efficiency and overall yield. Argon was also used with the same improvement in coupling efficiency, but argon was found to result in the introduction of bubbles into the exposure solvent in the reaction chamber during the light deprotection step, potentially resulting in significant additional light scattering. We used the 4-plex stepwise coupling microarrays to understand and quantify the effect of this drying step and to optimize its use. The value of the helium blow was first observed with via hybridization. Single-sequence microarrays with the 25mer sequence (see above) show an ~5-fold increase in hybridization signal when synthesized with a 30 second helium drying step between the acetonitrile washing step following coupling and the deprotection step. The synthesis protocol showing the timing of the drying step is shown in Table 1.

**Figure 5:**
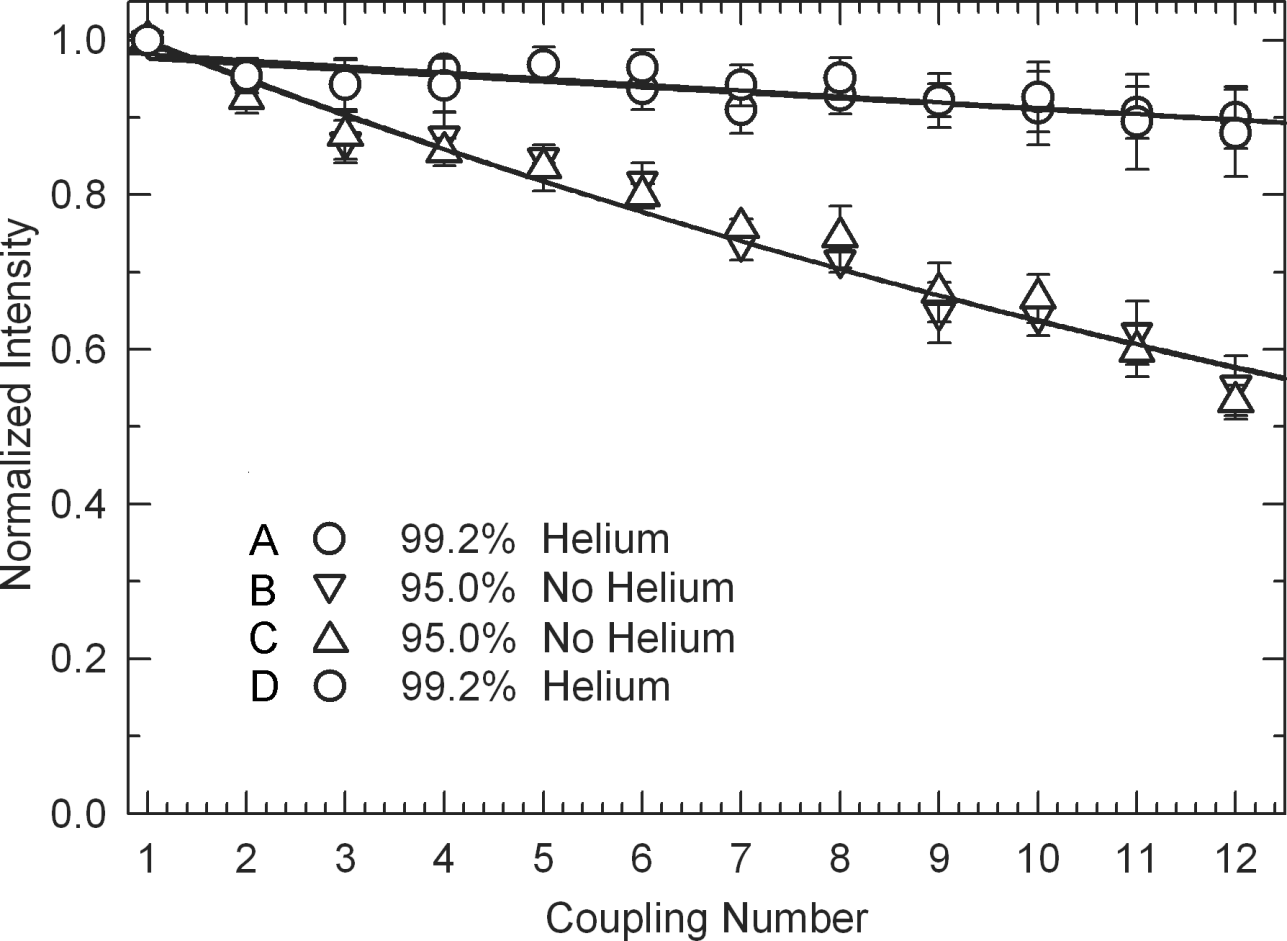
**Stepwise coupling efficiency of dT with or without a 30 s helium drying step following capping**. All of the plots are from data from a single 4-plex coupling microarray. The results show that the helium significantly increases coupling yield and that the helium step only increases the coupling efficiency of the immediately preceding coupling step. Percent coupling efficiency is derived from two parameter exponential fits of the data. Error bars are the standard deviation of the replicates.

Stepwise coupling efficiency measurement microarrays require capping after every coupling, but in the absence of an understanding of the mechanism, it was not initially clear if the helium drying step should be incorporated before or after capping. In the first experiment (see Table 2, Array 1), a 30 s helium drying step was inserted both between coupling and capping and between capping and light exposure, and compared with coupling efficiency on the same chip without any helium drying steps. Coupling efficiency with helium drying was much higher, 99.2% vs. 95.0%, as shown in Figure 5. One hypothesis was that the helium was facilitating photo-deprotection by clearing fluid out of the delivery and waste lines. The DMSO-based exposure solvent is significantly more viscous than the other synthesis solvents, ~2 cP vs. ~0.5 cP, and therefore flows more slowly through the synthesis cell, particularly when the waste line is full with the previously used solvent and reagent. To test this hypothesis, coupling efficiency was compared between coupling with the helium drying and coupling without the helium drying, but with higher delivery of exposure solvent. The rate of exposure solvent delivery was found not to be a factor (Table 2, Array 2). To see if the helium step was needed before or after capping, both options were tried on one array (Table 2, Array 3), which demonstrated that only drying the array after capping enhances coupling. A fourth experiment determined that 30 s is close to an optimal drying time (Table 2, Array 4). Figure 2 is a scan image of Array 4. Thin-film interference, visible to the unaided eye on the substrate during the drying step, indicates that 30 s of helium flow is the approximate amount of time needed to empty the synthesis cell and fully evaporate the final film of solvent on the glass substrate.

**Table 2:** Effect of drying microarray with helium.

Array 1	Helium before capping (s)	Helium after capping (s)	Coupling efficiency (%)
A	30	30	99.2
B	-	-	95.0
C	-	-	95.0
D	30	30	99.2

Array 2*			

A	30	30	98.4
B	30	30	98.4
C	-	-	94.1
D	-	-	93.8

Array 3			

A	15	-	95.6
B	-	30	98.6
C	30	-	95.7
D	30	30	98.4

Array 4			

A	30	30	99.3
B	-	10	99.0
C	-	30	99.3
D	-	60	99.3

Synthesis parameters: 12 J/cm^2^, 32 pulses exposure solvent*, 60 seconds coupling with 0.03 M NPPOC-T and 0.25 M DCI, and 120 seconds capping.

*Each sub-experiment of Array 2 uses a different number of pulses of exposure solvent during the 12 J/cm^2 ^exposure: (A) 32, (B) 60, (C) 60, and (D) 120.

Since the MAS starting point is a substrate with available hydroxyl groups, the coupling step precedes the deprotection step. This means that the deprotection step in each base protocol deprotects for the next coupling, e.g., the "A" synthesis protocol would typically deprotect for the "C" coupling, "C" for "G" and so forth. This means that the coupling experiments can determine if the helium blow is promoting coupling of the previous or the subsequent coupling. In all of the experiments, the drying step promotes coupling only in the preceding coupling step even though there is an intervening capping step and even though a drying step immediately after coupling has no effect. This suggests that the mechanism is related to the interaction between the exposure solvent and the preceding coupling reaction.

### Capping Efficiency

Acetic anhydride capping is useful in microarray-based experiments sensitive to coupling failures. Hybridization-based experiments are often not sensitive to this kind of error, but, for example, RNA-DNA chimeric microarrays used for studying enzyme kinetics [4] and microarrays used to generate oligonucleotides for gene assembly require effective capping to reduce the need for error correction at the data analysis stage. In addition, many of the experiments described herein, such as the coupling experiments, require effective capping. Capping efficiency is also a parameter used in the Monte Carlo simulations used to optimize synthesis and to interpret sequencing data from microarray-derived oligonucleotides (see below). Capping efficiency was measured using simple Cy3 end-labeled microarrays and standard MAS synthetic cycles (Table 1). After synthesis of a 15-mer thymidine linker, some parts of the microarray are deprotected with light (12 J/cm^2^, to reach almost complete photodeprotection), and then subject to a fake coupling with plain acetonitrile before capping. Other parts of the microarray receive a standard dT coupling cycle. Half of each of these two parts of the microarray are then deprotected and coupled with Cy3. The unlabeled areas are used for background subtraction. The capping efficiency calculated as: [I(fake_coupling_Cy3) - I(fake_coupling)]/[I(dT_Cy3) - I(dT)]. To reduce error from optical contrast effects, very large feature sizes were used in these experiments and fluorescence information from the scanned image was processed to remove pixel contributions originating from gaps between mirrors, where unintended exposure leads to significant Cy3 phosphoramidite coupling. In addition to acetic anhydride capping, phosphoramidite capping agents can be used. Unicap (diethyleneglycol ethyl ether (2-cyanoethyl)-(N, N-diisopropyl)-phosphoramidite) is available from Glen Research and has a reported efficiency of ~99%, but is fairly expensive. As an alternative, we have used 5'-dimethoxytrityl-dT phosphoramidite (DMT-dT) as an effective capping agent as previously reported by Chen et al. [28]. The dimethoxytrityl group is not removed by the photodeprotection step and DMT-dT is inexpensive and has a high coupling efficiency.

Table 3 shows the results of the capping efficiency experiments. For the acetic anhydride capping, only the total capping time was varied, with the volume of capping agents kept at a constant 20 pulses, 10 pulses to fill the flow cell followed by 10 more pulses over 30, 60 or 120 seconds (each Caps pulse on the Expedite 8909 consists of one pulse (~15 μL) of Cap A and one pulse of Cap B). Acetic anhydride capping kinetics are slow compared with phosphoramidite kinetics, with capping efficiency not reaching 95% until two minutes of exposure. For the DMT-dT capping experiments, the DMT-dT was coupled on the microarrays in the second of two consecutive coupling steps (the first six steps shown in Table 1). The first coupling step used plain acetonitrile instead of phosphoramidite to simulate synthesis with failed coupling, and the second coupled using 0.05 M DMT-dT in acetonitrile for either 15, 30 or 60 seconds. The acetic anhydride capping steps, which would usually follow coupling, were omitted, but the 30 second helium drying step was retained. Capping with DMT-dT is significantly faster than with acetic anhydride and results in more complete capping, with 97% efficiency achieved in 60 seconds. The efficiency of both capping methods is likely somewhat underestimated due photodeprotection less than 100% and incomplete removal of higher intensity scan pixels originating from mirror gaps. In addition, Cy3 surface quenching effects [25,29] which may disproportionally decrease fluorescence in the uncapped reference areas of the microarrays, would also lower the measured coupling efficiency. We estimate that these effects lower the measured capping efficiency by about two percentage points, indicating that the 60 seconds of DMT-dT capping approaches complete capping.

**Table 3:** Capping efficiency.

Agent*	Time (s)	Capping efficiency
Acetic anhydride	15	60 ± 4%
Acetic anhydride	30	81 ± 2%
Acetic anhydride	60	92 ± 2%
Acetic anhydride	120	95 ± 2%

DMT-dT	15	91 ± 2%
DMT-dT	30	94 ± 1%
DMT-dT	60	97 ± 1%

*Standard acetic anhydride capping: see Methods and Table 1 for capping protocol. For DMT-dT capping, the concentration was 0.05 M in acetonitrile and applied directly after coupling using the same pulse sequence as NPPOC amidite coupling.

### Optical contrast

Maskless array synthesis relies on the effective removal of the 5'-NPPOC hydroxyl protecting group of oligonucleotides prior to coupling a new base. The rate of photochemical removal of this group is directly proportional to light intensity and the number of remaining protecting groups:

(1) dσ(t)∕dt=-kIσ(t)

Where *σ*(*t*) is the concentration of yet-to-be-deprotected oligonucleotides at time *t *exposed to an intensity of light *I*. The rate constant *k *is empirically derived and depends on experimental parameters such as the wavelength of light and the ability of the exposure solvent to facilitate NPPOC removal. The solution to this simple differential equation is: *σ*(*t*) = σ_0 _exp(-*kIt*). Since the rate only depends on the concentration of one reactant, the protecting group, photodeprotection is a first order reaction [30]. This has significant implications for light-directed synthesis because unintended deprotection due to finite optical contrast cannot be avoided and leads to significant insertion errors [31].

**Figure 6:**
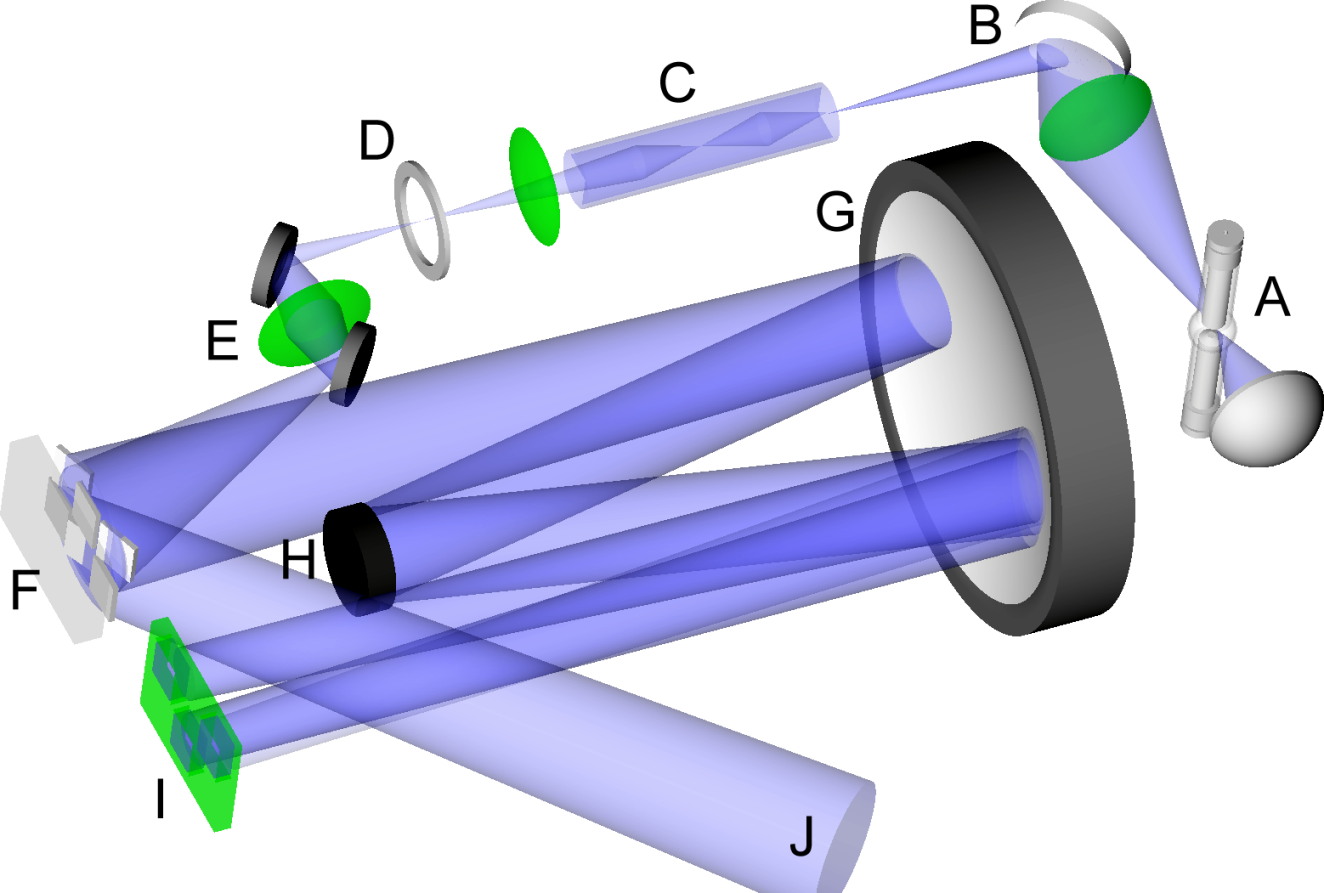
**Schematic of the optical system of the maskless array synthesizer**. A. High pressure mercury short-arc lamp. B. Dichroic mirror. C. Homogenizing light pipe. D. Shutter. E. Folding mirrors. F. Micromirror array. G. Offner relay primary mirror. H. Offner relay secondary mirror. I Reaction chamber illuminated by ON mirrors. J. Light dump from OFF mirrors.

**Figure 7:**
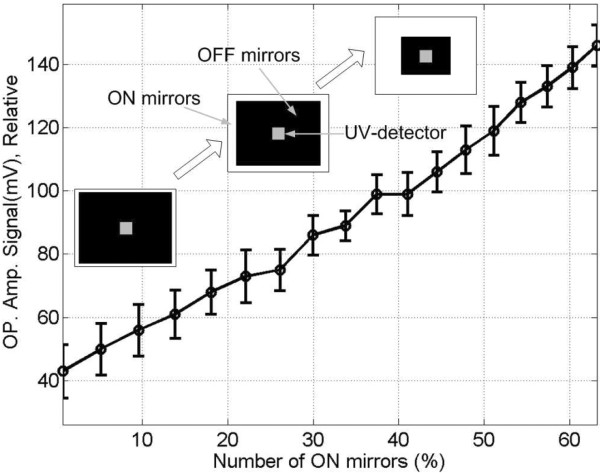
**Global flare is due to dust and imperfections of optical elements and leads to a homogenous background exposure of the synthesis surface**. With a UV intensity at the center of the image plane (gray squares in insets), global flare is measured from ON mirrors at the periphery of the DMD (white border of insets), which do not image directly into the detector. Global flare increases almost linearly with the number of ON mirrors. The central square in the inserts represents the photo-detector and the outer bands represent the number of ON mirrors. For 25% ON mirrors the irradiance (W/cm^2^) value is about 0.04% of direct irradiance.

A schematic of the optical system of the maskless array synthesizer is shown in Figure 6. Loss of optical contrast is mainly due to two factors, flare (scattering) and diffraction. Global flare originates from dust and imperfections in the optical system and results in a spatially homogenous background exposure that is proportional to the total number of ON mirrors as shown in Figure 7. In this experiment, a calibrated intensity meter is placed at the focal plane (replacing the synthesis cell), with a mask blocking all but a small central area of the light-sensitive surface as shown with the label "UV-detector" in Figure 7. With all the mirrors that direct light directly to the detector turned OFF, the measured signal is primarily from global flare irradiance originating from ON mirrors that do not image into the detector. At the high end of number of ON mirrors, 64%, we were able to measure a global flare irradiance of 0.1% of direct irradiance using a calibrated intensity meter. This corresponds to a global flare of less than 0.04% of synthesis irradiance for a 1:1 layout (all mirrors used) high density microarray, where an average of 25% of the mirrors are used in each deprotection step. This corresponds to a contrast ratio of better than 1/2500 (global flare only).

**Figure 8:**
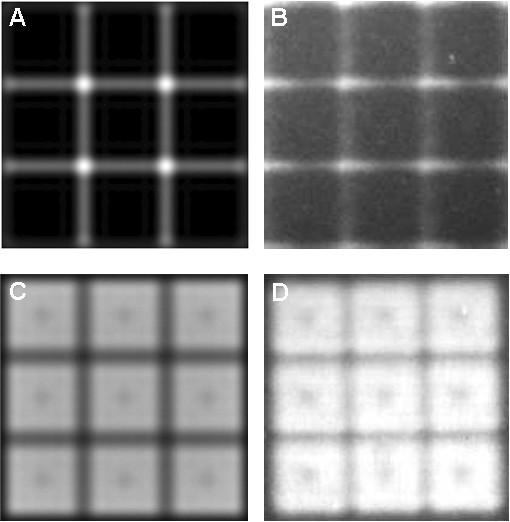
**Mirror-edge scattering is a form of local flare that is imaged to the synthetic surface and leads to exposure in the interstices between the synthesis pixels for both ON and OFF mirror positions**. A. Simulated edge scattering using Silvaco Optolith. B. Cy3-labeled, single dT microarray synthesized using long exposure only with OFF mirrors. C. Simulated exposure with ON mirrors. D. Cy3-labeled, single dT microarray synthesized using normal exposure with ON mirrors. The central dark regions are due to the mirror support post (part of the DMD mechanics), which both reduces mirror reflection in the center and introduces additional scattering.

**Figure 9:**
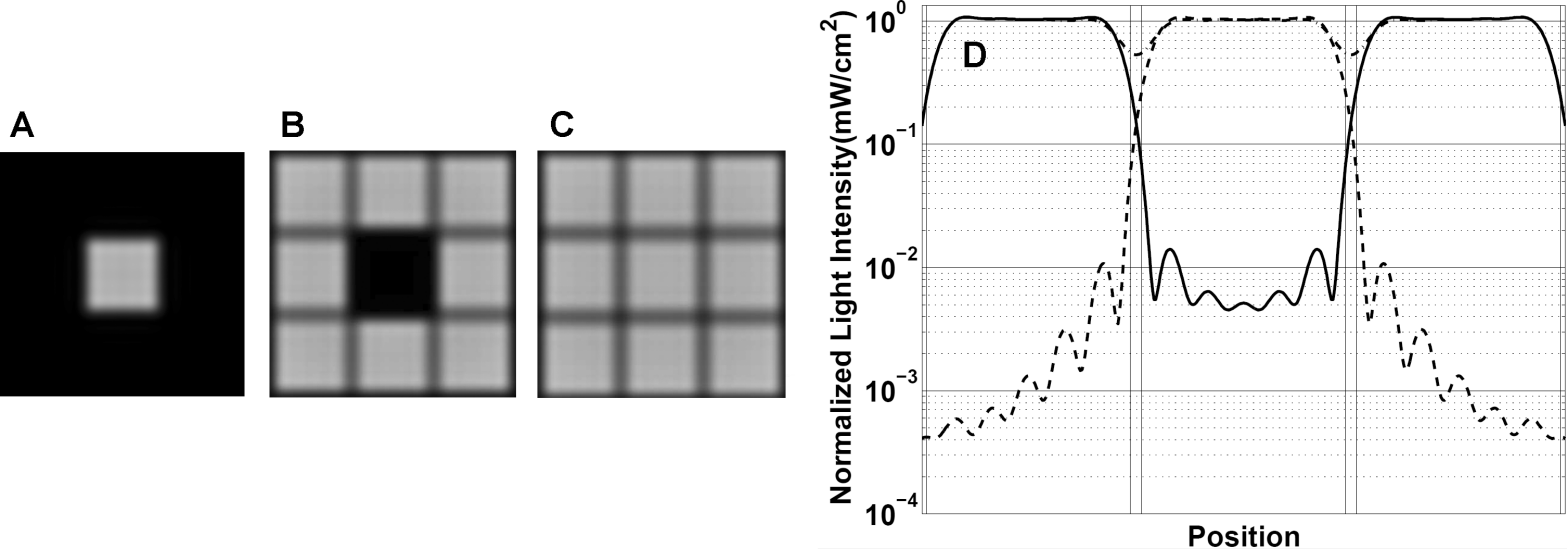
**Calculated aerial views of intensity on the synthetic surface from a 3 × 3 array of micromirrors**. A. Only central mirror ON. B. Only central mirror OFF. C. All mirrors ON. D shows a logarithmic plot of the light intensity reaching a central horizontal line through A (dash), B (solid) and C (dot-dash). In all cases the edges of the illuminated synthesis pixels lack sharpness, and particularly for the all ON pattern, the gaps between pixels are receive substantial intensity, about 50% of maximum pixel intensity.

Edge scattering originates from the micromirror edges (and to a much lesser extent, from the central mirror-support post) of both ON and OFF mirrors, and results in unintended exposure, primarily in the 1 μM gap between synthesis pixels (Figure 8). The magnitude of edge scattering is very low compared with direct irradiance, below the 0.1 W/cm^2 ^limit of detection of our calibrated UV meter, i.e., below 0.1% of the all mirrors ON direct irradiance of 100 W/cm^2^. Diffraction is an intrinsic limitation of all imaging systems and results in a superimposed pattern of light centered on each ON pixel. Diffraction results in loss of contrast at pixel edges and a pattern of partial exposure in adjacent pixels. Figure 9 shows calculated diffraction effects on a three-by-three grid of synthesis pixels with different patterns of ON and OFF mirrors: center ON, center OFF, and all ON. The primary consequence of diffraction is unintended exposure, ~10% of ON pixel exposure, in the gap between synthesis pixels and outer edge of adjacent pixels. The effects of both edge scattering and diffraction are primarily confined to interstices between the synthesis pixels. The sequences synthesized in these sites are random and short compared with the design sequences and do not hybridize significantly, but may result in additional purification requirements in gene assembly experiments.

Local flare is due to internal reflections along the optical path, in the synthesis cell, and from bubbles in the exposure solvent. Local flare is proportional to the density and proximity of synthesis pixels illuminated by ON mirrors. Local flare is the largest source of unintended exposure under most experimental conditions. Bubbles in the exposure solvent can contribute to local flare, but we have found that when helium (vs. argon) is used as to pressurize the synthesizer, bubbles are only produced in the first few hours after a new bottle of exposure solvent is added to the system. The primary source of local flare is back reflections along the light path, particularly from the surfaces of the quartz block. The back surface is coated with a broadband anti-reflection (BBAR) coating for the UV, but BBAR coatings reflect an average of ~0.25% of normal incident light (vs. ~4% for an uncoated glass-air interface), and significantly more if the surface is not clean and dust-free. In addition, there is a reflection from the exposure solvent (DMSO)-quartz interface at the back of the reaction cell. Even though DMSO has an index of refraction similar to that of quartz, they are not identical and the resulting reflection can be estimated at about 0.005% from the Fresnel equations and estimated values for the index of refraction of quartz (1.48) [32] and DMSO (1.50) [33] at 24°C and 365 nm. Reflections from the back surface of the quartz block appear to be the largest contributor to insertion errors within synthesis pixels. Diffraction exposure has a larger magnitude, but the exposure is primarily confined to the small area between synthesis pixels.

### Optimum dose

**Figure 10:**
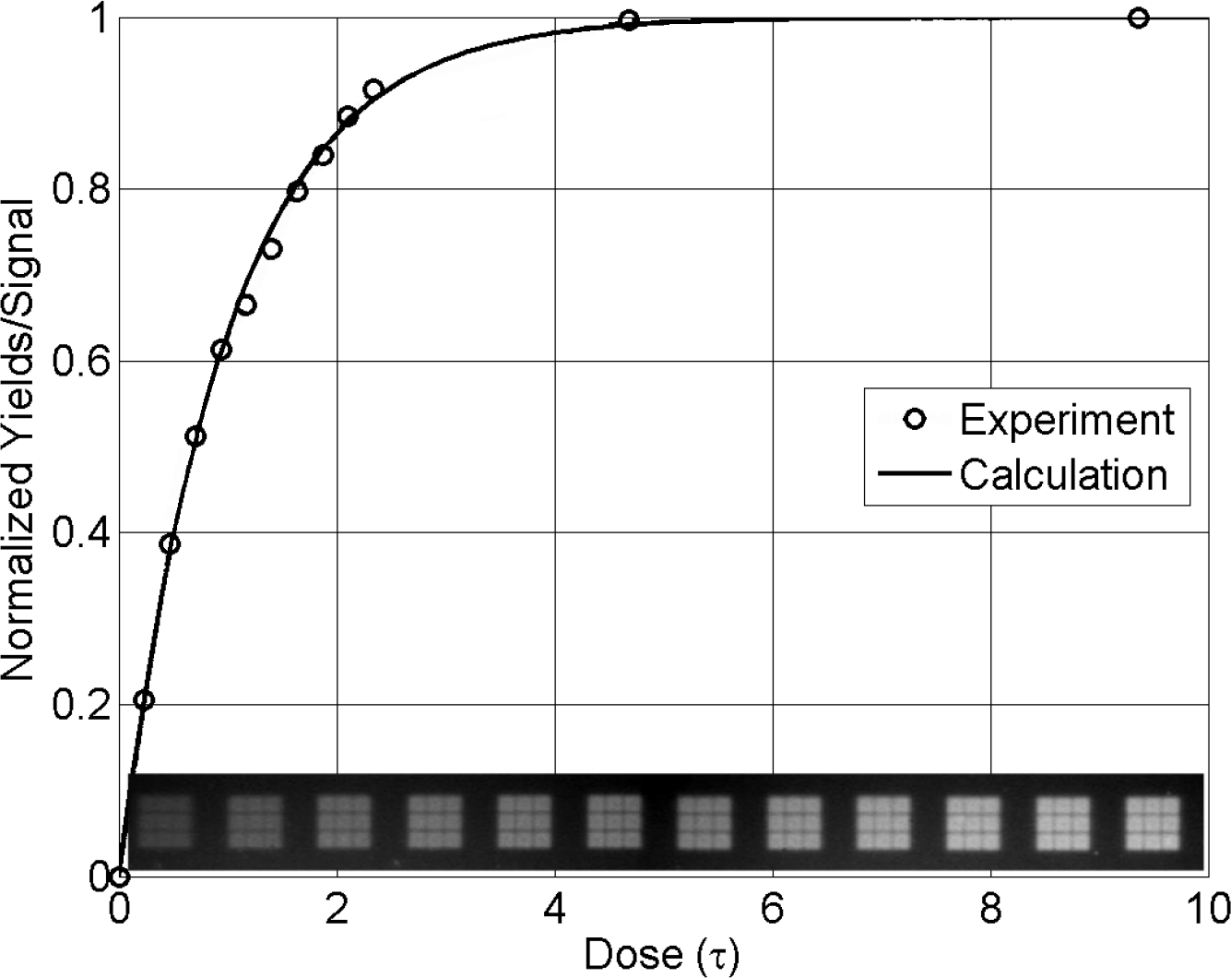
**Dose calibration curve using direct fluorescent end-labeling with Cy3**. A single base (dT) was coupled uniformly over the glass substrate and different areas (images at the bottom) were exposed to different doses of light before the final coupling with a Cy3 phosphoramidite. The time constant for photodeprotection *τ *equals approximately 2.5 J/cm^2^.

The optimal radiant exposure in the deprotection step in MAS is a compromise between using higher doses of light to minimize the number of deprotection failures and minimizing light to prevent insertion errors due to unintended exposure by scattered and diffracted light. Two methods were used for dose calibration, hybridization dose response and exposure gradients directly labeled with a fluorescent phosphoramidite. In both of these approaches, different doses are delivered to different areas of the microarray. In the case of directly labeling with Cy3, after an initial NPPOC-dT coupling, a single light gradient is applied prior to the Cy3 coupling. Figure 10 shows the result of this experiment. The normalized fluorescent intensity of the gradient follows the equation *F*(*t*) = 1 - exp(-*t*/*τ*), where *τ *(= *kI*, see above) is the time constant for photodeprotection. The dose for full (99.9%) deprotection is > 5*τ *corresponding to a radiant exposure of > 12 J/cm^2^. These values are based on light exposure using the I-line of mercury, as selected with 350-450 nm primary-range dichroic mirrors, and the use of 1% imidazole in DMSO to promote the pathway to NPPOC cleavage [34]. Hybridization-based dose response curves are synthesized in a similar way, but the photodeprotection gradient is repeated prior to each coupling, and the microarray is hybridized with a Cy3-labeled complementary sequence. Figure 4 shows several results from such hybridization gradients.

The optimal dose for hybridization-based experiments can be estimated directly from the curves in Figure 4. Although full deprotection is not realized, doses between 4 and 6 J/cm^2 ^result in close to the maximum of hybridization signal with a minimum of exposure. Compared with full photo-deprotection, this lower dose results in a lower surface density of hybridizable oligonucleotides, which increases the hybridization efficiency [28,35,36].

The optimal dose for gene assembly experiment must be calculated from the optical contrast data and confirmed with sequencing data from cleaved microarray oligonucleotides. Yield dependency on dose accounting for local optical contrast can be calculated from:

(2) $$Y\left(R,d\right)={\left(\gamma \delta_{\text{Bright}}\right)}^{N}{\left(1-\delta_{\text{Dark}}\right)}^{3N}$$

(3) $$\delta_{\text{Bright}}\left(d\right)=1-{\text{e}}^{-d∕\tau }$$

(4) $$\delta_{\text{Dark}}\left(d,R\right)=1-{\text{e}}^{-d∕\tau R}$$

**Figure 11:**
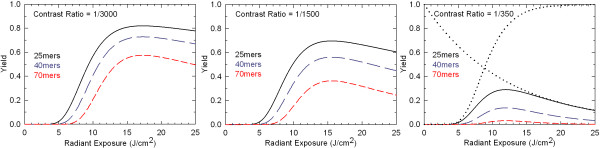
**Optimal photodeprotection dose for maximum correct sequence yield in microarray synthesis for gene assembly experiments**. The curves are calculated from Eqs. 2-4 with the coupling efficiency (*γ*) set to a value of 100% since coupling is an independent factor and does influence the calculation of the optimal dose. The two functions, bright and dark exposure deprotection yields, which are multiplied to give the correct sequence yield curve, are shown with dotted lines for the 25mer with contrast ratio of 1/350 in the right panel.

Where *γ *is the coupling efficiency, *d *is the dose in units of *τ*, *R *is the optical contrast, *N *is the oligonucleotide design length, and *δ*_Bright _and *δ*_Dark _are the deprotection yields for bright and dark exposure. Figure 11 shows the calculated results from Eqns. 2-4 for several values of optical contrast ratios and oligonucleotide lengths on the optimal photodeprotection dose.

Two types of microarray layout and protocols were used as sources of oligonucleotides for generating sequencing data. The first experiments were based on 80 different 70mer sequences per microarray, each synthesized in a large block, i.e., each of the sequences was synthesized in a large contiguous area of the microarray to minimize sequence error due to cross-contamination by stray light. Additional experimental parameters were: optimized set of 146 masks, no capping, and dose of 7*τ*. The sequencing data indicated an average deletion rate of 0.15 (1 in 7 bases), insertion rate of 0.036 (1 in 30 bases) and a perfect match yield of 2%. Monte Carlo simulations of the same data were used to extract additional synthesis parameters from a subset of the sequencing data: an average coupling efficiency of 98.3%, dose of 2.1*τ *and local optical contrast of 1/340. Sequences from microarrays made on a MAS instrument with extensive baffling against stray light had a deletion rate of 0.101 (1 in 10 bases), an insertion rate of 0.033 (1 in 30 bases) and a perfect match yield of 5%. Sequencing-based calibrations of optimal dose were performed on two types of microarray designs. The 60-mers on both arrays were synthesized with a "1-in-4" synthesis pixel layout and with different synthesis pixels on the same array receiving different doses. "1-in-4" layout means that only one-quarter of the DMD mirrors are used for synthesis, the unused mirrors result in one-mirror-sized margins with no DNA surrounding each microarray synthesis pixel. This layout is often used for high-density microarrays to avoid "cross-talk" between synthesis pixels when fluorescence intensity data is extracted using the common 5 μm-resolution scanners, which cannot resolve the gap between mirrors. The effects of stray light is reduced in this layout, compared with a "1-in-2" layout (checkerboard pattern) or "1-in-1" layout (all mirrors are used) at the expense of reduced array complexity.

The Chip-A design was synthesized without dark exposures (single sequence), while the Chip-B was synthesized with the 40 synthesis masks and 120 dummy exposures to simulate loss of optical contrast in normal multi-sequence microarray synthesis. For the Chip-A design, the resulting coupling efficiency was 98.5% and the dose response follows Eqn. 1 and the theoretical curve shown in Figure 10. The Chip-B leads to an optimal dose of 4.5*τ*, which, as expected, is lower than the saturating dose due to the optical contrast effects.

## Discussion

The stepwise coupling efficiency of the DNA phosphoramidites used in MAS is high, approaching the > 99% efficiency obtained with conventional solid phase synthesis. This is significantly higher that previously reported results for light-directed *in situ *microarray synthesis, in the range of 95% to 98% [25,37,38]. Some of the efficiency gains likely reflect improvements in coupling protocols and reagents, including reduced oxidation, helium drying, and the use of DCI as the activator. The stepwise coupling yield in MAS cannot be measured directly as in the case of trityl monitoring in solid phase synthesis. Because the stepwise coupling efficiency is significantly higher than the average stepwise yield calculated from the total fractional yield of full length oligonucleotides, previous results may have underestimated the stepwise coupling efficiency. Good coupling, in conjunction with the observed length-dependent loss of oligonucleotides during the coupling step, also indicates that the synthesis of long oligonucleotides with MAS benefit from shorter rather than longer coupling times, as well as optimizations of the synthesis protocol to minimize unnecessary exposure to the activator solution. An activator concentration lower than the current 0.25 M DCI may be beneficial, but this parameter has not yet been investigated. The oxidation requirement for MAS is also minimal because the array is not exposed to acidic deprotection solution, and a single final oxidation appears to be optimal. Capping may not be necessary for many hybridization-based applications of microarrays, but when capping is used, DMT-dT phosphoramidite is faster, more effective, and more economical than the conventional acetic anhydride reagents recommended for the Expedite synthesizers. Other acetic anhydride capping agents with higher concentrations of N-methylimidazole (MeIm) or with MeIm replaced by dimethylaminopyridine (DMAP) may also be more effective for MAS, but have not yet been tried.

Given the values for coupling efficiency that can currently be achieved in MAS, greater than ~98% for mixed sequence arrays of 40mers, optical contrast is approximately as important as coupling efficiency in determining sequence fidelity. Improvements in optical contrast are, therefore, means to improve the overall quality of MAS microarrays. The largest contributions to the measured value of the optical contrast ratio are diffraction and reflections from the back (air interface) side of the quartz block. Diffraction is an intrinsic limitation of any imaging system, but there are experimental strategies that can be applied to minimize the impact of diffraction on synthesis error. In the case of oligonucleotide synthesis for gene assembly, contiguous blocks of pixels synthesizing the same oligonucleotides increase the effective local contrast ratio because stray light from both diffraction and local flare primarily remains within the synthesis pixel block. Multi-mirror gaps ("streets") between synthesis blocks reduce stray-light cross-contamination. These streets can also be exposed and capped at the beginning of the synthesis to minimize oligonucleotide growth in these areas ("inverse capping") [39]. While pixel blocks are effective in reducing the effects of local stray light, diffraction and mirror gaps result in a spatially inhomogeneous dose within each block. The area corresponding to the gap between pixels receives the lowest dose, about 50% of the maximum, and the area corresponding to the outer edges of the mirror also receives a reduced dose (see Figure 9). Doubling the dose so that the areas corresponding to mirror gaps are fully deprotected doubles insertion errors due to global flare. While the contribution of global flare to insertion error would still be quite small, a better solution might be to slightly defocus the optics in order to homogenize the light intensity distribution at high spatial frequencies [40]. Although the use of multi-pixel blocks reduces the number of sequences that can be synthesized on the microarray, the gain in sequence fidelity is significant and is primarily limited by the global flare contrast ratio of ~1/2500.

For microarray applications where synthesizing the maximum number of sequences per microarray is desirable, such as gene expression experiments, sequence error due to diffraction is less problematic since hybridization of incorrect sequences is low. In addition, most of the incorrect sequences are located at the edges of each single-mirror feature, and these areas can be easily excluded from analysis at the stage when intensity data is extracted from the scan image. Reflection from the back side of the quartz block is the largest source of insertion error in the central areas of each single-mirror feature. Because of the thickness of this block, even a very small tilt relative to the optical axis will cause the refection to fall on adjacent synthesis pixels. Antireflective optical coatings reduce the contrast ratio contribution originating from this source of unintended exposure from ~1/25 to ~1/400. A simple avenue to significantly further reduce this number is the use of absorbent coatings applied to the backside of the quartz block, such as a refraction index-matched film containing dissolved molecules that strongly absorb near 365 nm.

## Conclusions

Recent improvements in maskless array synthesis phosphoramidite chemistry has resulted in stepwise coupling efficiencies comparable to solid-phase synthesis of oligonucleotides, ~99%. These efficiencies are significant improvement over early coupling results in light-directed synthesis of microarrays: 92-94% for 5'-MeNPOC-nucleoside phosphoramidites [25], and 96-99% for NPPOC phosphoramidites [37]. The increased coupling efficiency results in ~40-fold and ~2.5-fold full-length yield increases in the synthesis of 60mers, respectively, assuming a constant value of optical contrast. Compared with solid phase synthesis of single sequences, the approximate four-fold increase in the number of coupling steps required to synthesize the hundreds of thousands of unique sequences on a microarrays leads to a reduction of average coupling yield, from ~99% to ~98.5% for 40 mers. The source of this degradation has not yet been discovered, but can be reduced by shortening the coupling times to an optimal, probe-length-dependent compromise between stepwise coupling yield and sequence average yield of oligonucleotides. While coupling efficiency can likely be improved somewhat, at current coupling efficiency levels, stray light is the dominant source of sequence error in MAS. For conventional genomics applications based on microarray probe hybridization, stray-light induced sequence errors do not appear to play a significant role because most of the incorrect sequences are concentrated in the spaces between features (areas corresponding to gaps between mirrors, as well as unused mirrors) and do not participate in hybridization due to their short and quasi-random sequence. As an oligonucleotide source for gene assembly, MAS should be further optimized to increase the effective optical contrast. The extent to which improvements are necessary strongly depends on the sensitivity to sequence error of the gene assembly method. The dominant source of stray light is reflection from the back side of the reaction cell. Back reflections are easily addressed by absorptive coatings. The effects of stray light originating in scattering from mirrors and diffraction are also problematic for gene assembly experiments, but can be significantly reduced by straightforward experimental modifications such as inverse capping, the use of larger synthesis features, and slight defocusing of the imaging optics to homogenize the light intensity distribution at spatial frequencies corresponding to gaps between mirrors.

## Methods

All solvents and reagents were obtained from Sigma-Aldrich, unless otherwise noted, and used without further purification.

### Silanization of glass slides

Superclean SMC glass slides (ArrayIt, Sunnyvale, CA) were functionalized with a silane coupling agent, N-(3-triethoxysilylpropyl)-4-hydroxybutryamide (Gelest, Morrisville, PA), which functions as a linker and allows chemical cleavage of the oligonucleotides from the substrate [25]. The slides are placed in stainless steel rack and gently agitated in a solution of 2% (v/v) silane and 0.1% acetic acid in 95:5 ethanol/water. After 4 hour at room temperature, the slides are rinsed twice for 15 min in the aqueous ethanol and allowed to cure for several hours at 120°C under vacuum. After cooling to room temperature under vacuum, the slides are stored in a desiccator until use.

### Optical system

**Figure 12:**
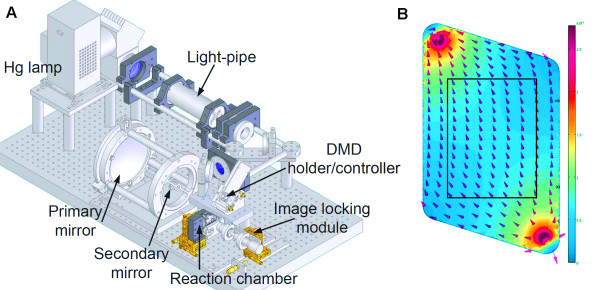
**A 3-D visualization of the MAS optical system with labels indicating the major components**. B. Synthesis reaction chamber with arrows and colors indicating relative reagent velocity within the parallelogram-shaped volume defined by the gasket and across the microarray surface (central rectangle). The reagent flow pattern indicates that the cell geometry prevents stagnant zones which might hinder array uniformity. Relative to the illustration, when mounted in the synthesizer, the reaction chamber is rotated by 45° clockwise, with reagents entering at the bottom and exiting at the top (black spots). The scale of the chamber is defined by the rectangle, which, due to the 1:1 imaging system has the same dimensions as the DMD (14 × 10 mm).

The maskless array synthesizer consists of an optical imaging system and a reagent delivery system. In the optical system, shown schematically in Figure 12A, light from a 350 W Hg short arc lamp (Newport, Stratford, CT) is filtered with two 350-450 nm primary-range dichroic mirrors (Newport) and directed into a square cross section light pipe [41], which spatially homogenizes the irradiance distribution and shapes the beam to fit the digital micromirror device (Texas Instruments 0.7 XGA DMD). Light exiting the light pipe is directed by two folding mirrors through two lenses and a shutter to the DMD. Each of the mirrors in the DMD is independently addressable and has two positions, one directs light further through the optical system (ON), the other to an absorbing surface (OFF). The ON pattern on the DMD is projected to the synthesis substrate with an Offner relay system, a 1:1 imaging design that provides a high image quality with simple optics [42,43]. The display of virtual masks on the DMD and the opening of the shutter are controlled by an external computer. The computer also receives a signal from the oligonucleotide synthesizer which permits the synchronization of the light deprotection with the phosphoramidite chemistry on the microarray surface.

The substrate is secured to a quartz block with a perfluoroelastomer gasket to form a ~100 μm deep reaction chamber with a volume of 45 μL. Two holes in the quartz block, an inlet and an outlet, are arranged to allow reagents to uniformly cover the microarray (Figure 12B). The reaction chamber is held together and supported by an aluminum mount that can be precisely positioned in the optical system. The reaction chamber is connected to the Expedite 8909 by 405 μm inner diameter fluorinated ethylene propylene (FEP) tubing (IDEX Health and Science part number 1692). The reagent flow volumes given below are based on an inlet tubing length of 68 cm. The reaction chamber mount is normally positioned with the glass substrate facing the DMD and with the inner glass surface at the image plane. The back side of the quartz block has an antireflection coating to reduce unintended deprotection. For opaque substrates, the reaction chamber mount can be reversed, with the quartz block functioning as the window into the reaction chamber.

### Chemical synthesis

*In situ *microarray synthesis phosphoramidite chemistry is similar to that used in solid-phase synthesis of single-sequence oligonucleotides. Since the substrate originally has, or is functionalized to have, reactive hydroxyl groups, the synthetic cycle follows the following order: (1) coupling, (2) capping, (3) photo-deprotection. An oxidation step (tetrahydrofurane/water/pyridine/iodine 66/12/22/0.6 (v/v/v/m)) is not required in each cycle because the light deprotection step does not cleave the oligonucleotides at the phosphite ester bonds, allowing periodic or a single final oxidation step. Table 1 shows representative chemical protocols. Reagents are delivered to the reaction cell by a Perspective Biosystems Expedite 8909 oligonucleotide synthesizer. The Expedite's Luer taper fittings for attaching to controlled pore glass columns are replaced with threaded fittings that connect with tubing leading to the reaction cell.

The coupling reaction proceeds by first filling the synthesis cell with activator (typically 0.25 M 4,5-dicyanoimidazole (DCI) in acetonitrile), followed by a 1:1 mix of activator and the desired phosphoramidite solution. The phosphoramidites for MAS have the 2-(2-nitrophenyl)propyloxycarbonyl (NPPOC) photocleavable 5'-hydroxyl protecting group and the standard 3'-β-cyanoethyl group. The phosphoramidites are dissolved in dry acetonitrile (< 30 ppm water) to a concentration of 0.02 to 0.03 M.

After coupling, the array is washed with acetonitrile. If capping is used to truncate polymerization after failed couplings, the reaction cell is filled with a 1:1 mix of tertbutylphenoxyacetyl acetic anhydride (Tac_2_O) in tetrahydrofuran (Cap A) and 10% N-methylimidazole in tetrahydrofuran/pyridine (8:1) (Cap B). Following capping, the reaction cell is dried with helium. If capping is not used, the reaction cell is dried directly after each coupling.

The deprotection step characterizes MAS and consists of exposing selected features of the microarrays to near ultraviolet light to remove the 5'-NPPOC group, exposing the terminal hydroxyls to which the phosphoramidites should couple in the next cycle. During the exposure, the array is washed with an exposure solvent which includes an organic base, usually a non-nucleophilic heterocyclic secondary or tertiary amine [44,45]. The base promotes the chemical pathway to cleavage of the NPPOC after it has absorbed a photon [34]; one percent (m/v) imidazole in anhydrous dimethyl sulfoxide (DMSO) works well; 25 mM piperidine in acetonitrile has also been used successfully [46]. The DMSO has an index of refraction similar to that of quartz and glass, which reduces light reflection and scattering at the respective reaction cell interfaces.

The irradiance of UV light reaching the synthesis surface is determined with a probe positioned at the image plane. We use a SUSS Model 1000 UV intensity meter with a 365 nm probe (SÜSS MicroTec, Waterbury Center, VT). Once the irradiance (W/cm^2^) is determined, the length of the exposure can be calculated based on the desired radiant exposure (J/cm^2^). The irradiation is a critical parameter that needs to be balanced between maximum deprotection and minimum scattering and flare. A typical irradiance of 100 mW/cm^2^, which can be obtained for most of the lifetime of the mercury lamp, results in an irradiation of 6 J/cm^2 ^in 60 seconds, a typical value used for exposures of oligonucleotide microarrays intended for hybridization- or protein-binding-based experiments.

After microarray synthesis, the base and phosphate protecting groups need to be removed to generate true DNA. For this purpose, the array-bearing substrate is immersed in 1:1 (v/v) ethylenediamine/ethanol for two hours at room temperature, then washed twice in deionized water and dried by blowing with a clean compressed gas. If the microarray is terminally labeled (e.g., Cy3, Cy5, or biotin phosphoramidites; Glen Research, Sterling, VA), it is washed vigorously, after synthesis, for about 2 hours in acetonitrile to remove dye molecules non-specifically bound to the oligonucleotides or the substrate.

### Hybridization

To determine the optimal synthesis parameters for hybridization-based microarray experiments, test microarrays were hybridized in a manner representative of genomics hybridization experiments. First the microarrays are blocked for 15 min at 45°C with 40 μg herring sperm DNA and 200 μg acetylated BSA in 400 μL MES buffer (100 mM MES, 1 M Na^+^, 20 mM EDTA, 0.01% Tween20) using an adhesive hybridization chamber (SecureSeal SA200, Grace Bio-labs). After removing the blocking buffer, the microarrays were incubated with a similar buffer with Cy3-labeled probe oligonucleotides at a concentration of 10 nM. An air bubble of about ¼ the chamber volume aids mixing during hybridization [47]. After 2 hrs of rotation in a hybridization oven, the chamber was removed and the microarrays were vigorously washed in a 50 ml centrifuge tube with non-stringent wash buffer (SSPE; 0.9 M NaCl, 0.06 M phosphate, 6 mM EDTA, 0.01% Tween20) for 2 min. and then with stringent wash buffer (100 mM MES, 0.1 M Na^+^, 0.01% Tween20) for 1 min. Finally, the microarrays were dipped for a few seconds in final wash buffer, either NimbleGen final wash buffer (Wash buffer III) or 0.1× saline-sodium citrate (SSC) buffer, and then dried with argon.

### Microarray imaging and data extraction

All microarrays were scanned with either a Molecular Devices GenePix 4000B or 4100A, or an Applied Precision Microarray Scanner. Data was extracted using either GenePix Pro software, or in the case of high-density microarrays, with NimbleScan (Roche-NimbleGen).

### Sequencing

MAS sequencing data was obtained from specifically designed microarrays. Each microarray consisted of either a 8 × 10 grid made up from 100 × 100 synthesis pixels (a pixel refers to the synthesis area corresponding to a single micromirror), or a 1 in 4 layout. A unique 70-mer was synthesized on each grid element. Each 70-mer consisted of a 40-mer experimental sequence flanked by 15-mer PCR primers. After synthesis, the oligonucleotides were cleaved from the substrate with NH_4_OH for 1 h at room temperature. Base-protecting groups were removed from the eluted oligonucleotides in NH_4_OH for 16 h [48]. The eluate was then dried in a vacuum centrifuge and re-suspended in deionized water. The oligonucleotides were then PCR-amplified, cloned and sequenced using a Genome Sequencer 20 from 454 Life Sciences.

### Simulations

In order to optimize oligonucleotide synthesis, all imaging details, in addition to chemical kinetics, need to be included in a synthesis model encompassing diffraction, flare, and optical system parameters. Comparison of experimental and simulation data can also assist system debugging and optical/chemical optimization. Hence, we developed a comprehensive photon-based stochastic 2D Monte-Carlo (MC) simulation [49] and applied it to MAS synthesis for understanding the sequence formation mechanism further and for extracting critical synthesis parameters for further optimization. Two dimensional aerial image patterns (light intensity maps) at the synthesis sites formed by a given mask pattern were generated by Optolith (Silvaco, Santa Clara, CA). This optical lithography tool models the transfer of the mask pattern (here, the mirror array) onto the substrate, taking into account the optical parameters of the imaging system: NA = 0.08, σ = 0 (zero partial coherence with a light uniformizer in place, otherwise 0.7) and λ = 365 nm. Figure 9 A, B, C show the Optolith grayscale image output for three different microarray layouts, and Figure 9D is a plot of the normalized intensity through a center line. To simulate global flare originating from all of the mirrors of the DMD outside the Optolith model, a constant level of extrinsic flare is added to the light intensity map by uniformly increasing the intensity of each pixel of the grayscale image. Due to the calculation load, the Optolith simulation was done at sparser grids than the actual reaction sites. Therefore, the light-intensity map was expanded to the actual reaction site density with a bilinear interpolation using Matlab. The resulting light intensity map contains optical diffraction and flare information. The fidelity of calculated aerial image was experimentally verified to be the same as actual images, as shown in Figure 8 and in references [31,38]. The intensity distribution obtained from the Optolith simulation was converted to photon arrivals at discrete reaction site coordinates using an inverse transform method [49]. Photodeprotection events were assigned to oligonucleotides based on the combination of a single photon arrivals and the empirical photochemical quantum efficiency of the deprotection reaction. Phosphoramidite coupling, acetic anhydride capping events were randomly assigned via their efficiencies based on zero order chemical reaction kinetics. Oxidation was assumed to be 100% efficient. The DNA sequence of each reaction site was based on the four reaction events per cycle and 146 cycles (optimized mask set for the core 40-mer). At the completion of the simulation, the entire set of the sequences of oligonucleotides in the region of simulation are ready for further analysis and can be compared to experimental sequencing data. The full details of the Monte Carlo simulations and data analysis are available in ref. [50].

## List of Abbreviations

BSA: Bovine serum albumin; Cap A: Tertbutylphenoxyacetyl acetic anhydride in tetrahydrofuran (1:1); Cap B: 10% N-methylimidazole in tetrahydrofuran/pyridine (8:1); DCI: 4,5-dicyanoimidazole; DMD: Digital micromirror device; DMT: Dimethoxytrityl; NPPOC: 2-(2-nitrophenyl)propyloxycarbonyl; MAS: Maskless array synthesis; MES 2-(N-morpholino)ethanesulfonic acid; SSC: Saline-sodium citrate; Tac_2_O: Tertbutylphenoxyacetyl acetic anhydride; XGA: Extended graphics array.

## Competing interests

The authors declare that they have no competing interests.

## Authors' contributions

All of the authors contributed substantially by performing experiments, computer simulations, analyzing data and/or writing/improving the hardware/software. MS wrote the manuscript with significant contributions from CK. All authors read and approved the final manuscript.

## References

[B1] Fodor S Read J Pirrung M Stryer L Lu A Solas D Light-directed, spatially addressable parallel chemical synthesis Science 1991 251 767 773 10.1126/science.1990438 1990438

[B2] Singh-Gasson S Green RD Yue YJ Nelson C Blattner F Sussman MR Cerrina F Maskless fabrication of light-directed oligonucleotide microarrays using a digital micromirror array Nature Biotechnology 1999 17 974 978 10.1038/13664 10504697

[B3] Lackey JG Mitra D Somoza MM Cerrina F Damha MJ Acetal Levulinyl Ester (ALE) Groups for 2'-Hydroxyl Protection of Ribonucleosides in the Synthesis of Oligoribonucleotides on Glass and Microarrays Journal of the American Chemical Society 2009 131 8496 8502 10.1021/ja9002074 19485360

[B4] Lackey JG Somoza MM Mitra D Cerrina F Damha MJ In-situ chemical synthesis of rU-DNA chimeras on chips and enzymatic recognition Chim Oggi-Chem Today 2009 27 30 33

[B5] Pellois JP Zhou X Srivannavit O Zhou T Gulari E Gao X Individually addressable parallel peptide synthesis on microchips 2002 20 922 926 10.1038/nbt723 12134169

[B6] Shin D-S Lee K-N Yoo B-W Kim J Kim M Kim Y-K Lee Y-S Automated Maskless Photolithography System for Peptide Microarray Synthesis on a Chip Journal of Combinatorial Chemistry 2010 12 463 471 10.1021/cc100009g 20666398

[B7] Phillips MF Lockett MR Rodesch MJ Shortreed MR Cerrina F Smith LM In situ oligonucleotide synthesis on carbon materials: stable substrates for microarray fabrication Nucleic Acids Research 2008 36 e7 10.1093/nar/gkm1103 PMC2248760 18084027

[B8] Lockett MR Weibel SC Phillips MF Shortreed MR Sun B Corn RM Hamers RJ Cerrina F Smith LM Carbon-on-Metal Films for Surface Plasmon Resonance Detection of DNA Arrays Journal of the American Chemical Society 2008 130 8611 8613 10.1021/ja802454c PMC2527731 18597426

[B9] Negrete OD Onses MS Nealey PF Cerrina F In situ synthesis and direct immobilization of ssDNA on electron beam patterned hydrogen silsesquioxane J Vac Sci Technol B 2009 27 3082 3087

[B10] Pon RT Yu S Tandem oligonucleotide synthesis using linker phosphoramidites Nucleic Acids Research 2005 33 1940 1948 10.1093/nar/gki333 PMC1074720 15814811

[B11] Wang T Oehrlein S Somoza MM Perez JRS Kershner R Cerrina F Optical tweezers directed one-bead one-sequence synthesis of oligonucleotides Lab Chip 2011 11 1629 1637 10.1039/c0lc00577k 21445444

[B12] Lemoine S Combes F Le Crom S An evaluation of custom microarray applications: the oligonucleotide design challenge Nucleic Acids Research 2009 37 1726 1739 10.1093/nar/gkp053 PMC2665234 19208645

[B13] Relogio A Schwager C Richter A Ansorge W Valcarcel J Optimization of oligonucleotide-based DNA microarrays Nucleic Acids Research 2002 30 e51 10.1093/nar/30.11.e51 PMC117213 12034852

[B14] Chou C-C Chen C-H Lee T-T Peck K Optimization of probe length and the number of probes per gene for optimal microarray analysis of gene expression Nucleic Acids Research 2004 32 e99 10.1093/nar/gnh099 PMC484198 15243142

[B15] Dill K, Liu R, Grodzinsky P Microarrays. Preparation, Microfluidics, Detection Methods, and Biological Applications 2009 Springer

[B16] Carlson CD Warren CL Hauschild KE Ozers MS Qadir N Bhimsaria D Lee Y Cerrina F Ansari AZ Specificity landscapes of DNA binding molecules elucidate biological function Proceedings of the National Academy of Sciences 2010 107 4544 4549 10.1073/pnas.0914023107 PMC2842033 20176964

[B17] Warren CL Kratochvil NCS Hauschild KE Foister S Brezinski ML Dervan PB Phillips GN Ansari AZ Defining the sequence-recognition profile of DNA-binding molecules Proceedings of the National Academy of Sciences of the United States of America 2006 103 867 872 10.1073/pnas.0509843102 PMC1347994 16418267

[B18] Puckett JW Muzikar KA Tietjen J Warren CL Ansari AZ Dervan PB Quantitative Microarray Profiling of DNA-Binding Molecules Journal of the American Chemical Society 2007 129 12310 12319 10.1021/ja0744899 PMC3066056 17880081

[B19] Tian J Gong H Sheng N Zhou X Gulari E Gao X Church G Accurate multiplex gene synthesis from programmable DNA microchips 2004 432 1050 1054 10.1038/nature03151 15616567

[B20] Binkowski BF Richmond KE Kaysen J Sussman MR Belshaw PJ Correcting errors in synthetic DNA through consensus shuffling Nucleic Acids Research 2005 33 e55 10.1093/nar/gni053 PMC1072806 15800206

[B21] Carr PA Park JS Lee Y-J Yu T Zhang S Jacobson JM Protein-mediated error correction for de novo DNA synthesis Nucleic Acids Research 2004 32 e162 10.1093/nar/gnh160 PMC534640 15561997

[B22] Smith HO Hutchison CA Pfannkoch C Venter JC Generating a synthetic genome by whole genome assembly:ΦX174 bacteriophage from synthetic oligonucleotides Proceedings of the National Academy of Sciences of the United States of America 2003 100 15440 15445 10.1073/pnas.2237126100 PMC307586 14657399

[B23] Septak M Kinetic Studies on Depurination and Detritylation of CPG-Bound Intermediates During Oligonucleotide Synthesis Nucleic Acids Research 1996 24 3053 3058 10.1093/nar/24.15.3053 PMC146050 8760893

[B24] Garland PB Serafinowski PJ Effects of stray light on the fidelity of photodirected oligonucleotide array synthesis Nucleic Acids Research 2002 30 e99 10.1093/nar/gnf098 PMC140559 12364616

[B25] McGall GH Barone AD Diggelmann M Fodor SPA Gentalen E Ngo N The Efficiency of Light-Directed Synthesis of DNA Arrays on Glass Substrates Journal of the American Chemical Society 1997 119 5081 5090

[B26] Agbavwe C Somoza MM Sequence-Dependent Fluorescence of Cyanine Dyes on Microarrays PLoS ONE 2011 6 e22177 10.1371/journal.pone.0022177 PMC3143128 21799789

[B27] Pon RT Damha MJ Ogilvie KK Modification of guanine bases by nucleoside phosphoramidite reagents during the solid phase synthesis of oligonucleotides Nucleic Acids Research 1985 13 6447 6465 10.1093/nar/13.18.6447 PMC321970 4059050

[B28] Chen S Phillips MF Cerrina F Smith LM Controlling Oligonucleotide Surface Density in Light-Directed DNA Array Fabrication Langmuir 2009 25 6570 6575 10.1021/la9000297 PMC2693259 19281155

[B29] Beier M Hoheisel JD Production by quantitative photolithographic synthesis of individually quality checked DNA microarrays Nucleic Acids Research 2000 28 e11 10.1093/nar/28.4.e11 PMC102590 10648799

[B30] Wöll D Walbert S Stengele K-P Albert TJ Richmond T Norton J Singer M Green RD Pfleiderer W Steiner UE Triplet-Sensitized Photodeprotection of Oligonucleotides in Solution and on Microarray Chips Helv Chim Acta 2004 87 28 45

[B31] Kim C Li M Lowe A Venkataramaiah N Richmond K Kaysen J Cerrina F DNA microarrays: An imaging study Journal of Vacuum Science & Technology B: Microelectronics and Nanometer Structures 2003 21 2946 2950

[B32] Rodney WS Spindler RJ Index of Refraction of Fused Quartz Glass for Ultraviolet, Visible, and Infrared Wavelengths Journal of the Optical Society of America 1954 44 677 678

[B33] Pacak P Polarizability and molecular radius of dimethyl-sulfoxide and dimethylformamide from refractive index data Book Polarizability and molecular radius of dimethyl-sulfoxide and dimethylformamide from refractive index data 1987 16 City: Springer Netherlands 71 77

[B34] Walbert S Pfleiderer W Steiner UE Photolabile Protecting Groups for Nucleosides: Mechanistic Studies of the 2-(2-Nitrophenyl)ethyl Group Helv Chim Acta 2001 84 1601 1611

[B35] Gao Y Wolf LK Georgiadis RM Secondary structure effects on DNA hybridization kinetics: a solution versus surface comparison Nucleic Acids Research 2006 34 3370 3377 10.1093/nar/gkl422 PMC1488884 16822858

[B36] Peterson AW Heaton RJ Georgiadis RM The effect of surface probe density on DNA hybridization Nucleic Acids Research 2001 29 5163 5168 10.1093/nar/29.24.5163 PMC97561 11812850

[B37] Nuwaysir EF Huang W Albert TJ Singh J Nuwaysir K Pitas A Richmond T Gorski T Berg JP Ballin J Gene Expression Analysis Using Oligonucleotide Arrays Produced by Maskless Photolithography Genome Research 2002 12 1749 1755 10.1101/gr.362402 PMC187555 12421762

[B38] Kim C Li M Rodesch M Lowe A Richmond K Cerrina F Biological lithography: Improvements in DNA synthesis methods J Vac Sci Technol B 2004 22 3163 3167

[B39] Kim C Kaysen J Richmond K Rodesch M Binkowski B Chu L Li M Heinrich K Blair S Belshaw P Progress in gene assembly from a MAS-driven DNA microarray Microelectronic Engineering 2006 83 1613 1616

[B40] Offner A Wavelength and coherence effects on the performance of real optical projection systems Photogr Sci Eng 1979 23 374

[B41] Cheng Y-K Chern J-L Irradiance formations in hollow straight light pipes with square and circular shapes Journal of the Optical Society of America A 2006 23 427 434 10.1364/josaa.23.000427 16477846

[B42] Suzuki A Complete analysis of a two-mirror unit magnification system. Part 1 Applied Optics 1983 22 3943 3949 10.1364/ao.22.003943 18200292

[B43] Offner A New concepts in projection mask aligners Optical Engineering 1975 14 130 132

[B44] Beier M Matysiak S Hoheisel J Method for the light-controlled synthesis of biochips Book Method for the light-controlled synthesis of biochips 2004 City: Deutsches Krebsforschungszentrum Stiftung des öffentlichen Rechts

[B45] Pirrung MC Wang L Montague-Smith MP 3'-Nitrophenylpropyloxycarbonyl (NPPOC) Protecting Groups for High-Fidelity Automated 5' to' 3' Photochemical DNA Synthesis Organic Letters 2001 3 1105 1108 10.1021/ol0069150 11348170

[B46] Naiser T Ehler O Kayser J Mai T Michel W Ott A Impact of point-mutations on the hybridization affinity of surface-bound DNA/DNA and RNA/DNA oligonucleotide-duplexes: Comparison of single base mismatches and base bulges BMC Biotechnology 2008 8 48 10.1186/1472-6750-8-48 PMC2435543 18477387

[B47] McQuain MK Seale K Peek J Fisher TS Levy S Stremler MA Haselton FR Chaotic mixer improves microarray hybridization Analytical Biochemistry 2004 325 215 226 10.1016/j.ab.2003.10.032 14751256

[B48] Richmond KE Li M-H Rodesch MJ Patel M Lowe AM Kim C Chu LL Venkataramaian N Flickinger SF Kaysen J Amplification and assembly of chip-eluted DNA (AACED): a method for high-throughput gene synthesis Nucleic Acids Research 2004 32 5011 5018 10.1093/nar/gkh793 PMC521639 15448182

[B49] Rubinstein RY Simulation and the Monte Carlo Method 1981 John Wiley & Sons

[B50] Kim C Gene Synthesis from Photolithographic DNA Microarrays Ph.D. thesis 2007 University of Wisconsin, Electrical Engineering

